# Nanoplastics Elicit Stage-Specific Physiological, Biochemical, and Gut Microbiome Responses in a Freshwater Mussel

**DOI:** 10.3390/toxics13050374

**Published:** 2025-05-05

**Authors:** Yangli Chi, Hui Zhang, Jian Gao, Liang Wan, Yiying Jiao, Heyun Wang, Mingjun Liao, Ross N. Cuthbert

**Affiliations:** 1 Key Laboratory of Intelligent Health Perception and Ecological Restoration of Rivers and Lakes, Ministry of Education, Hubei University of Technology, Wuhan 430068, China; 15002778975@163.com (Y.C.); huizhangeco@163.com (H.Z.); liangwan@hbut.edu.cn (L.W.); jiaoyiying1223@hbut.edu.cn (Y.J.); heyunwang2006@163.com (H.W.); lmj1112@163.com (M.L.); 2 Hubei Key Laboratory of Environmental Geotechnology and Ecological Remediation for Lake and River, Innovation Demonstration Base of Ecological Environment Geotechnical and Ecological Restoration of Rivers and Lakes, Hubei University of Technology, Wuhan 430068, China; 3 Institute for Global Food Security, School of Biological Sciences, Queens University Belfast, Belfast BT9 5DL, UK; r.cuthbert@qub.ac.uk

**Keywords:** nanoplastics, *Cristaria plicata*, intestinal gland, oxidative stress, physiological metabolism, gut microbiota

## Abstract

Mussels are highly efficient filter feeders, playing a crucial role in managing eutrophication and assessing pollution. Although research on nanoplastic (NP) toxicity in marine organisms is expanding, studies on freshwater species remain limited despite freshwater ecosystems being disproportionately biodiverse and vulnerable to pollutants. Here, we quantified the effects of polystyrene nanoplastics (PS-NPs, 50 nm) at concentrations of 0, 2, 20, and 200 μg/L on different growth stages of the freshwater mussel *Cristaria plicata*. After a 45-day exposure, PS-NPs at concentrations ≥ 20 μg/L damaged intestinal epithelial cilia in both age groups. Exposure to 200 μg/L PS-NPs significantly increased malondialdehyde levels and decreased superoxide dismutase activity in both groups, with adults showing a significant rise in total protein content and juveniles exhibiting marked increases in respiratory and ammonia excretion rates. Additionally, PS-NP exposure significantly altered the relative abundance of gut microbial phyla, including Proteobacteria, Firmicutes, Verrucomicrobiota, and Bacteroidota, with Fusobacteriota also being affected in adults. Juveniles were more sensitive to physiological changes, whereas adults exhibited greater microbiota shifts in response to PS-NP exposure. Therefore, this study provides new insights into the stage-specific effects of PS-NPs on intestinal integrity and physiological and biochemical health in freshwater mussels, underscoring the need for targeted management strategies to protect freshwater ecosystems.

## 1. Introduction

Freshwater mussels (Bivalvia, Unionoida) are highly efficient filter feeders that play vital roles in freshwater ecosystems [1,2,3]. By filtering water and sediments, they effectively remove bacteria, phytoplankton, and dissolved organic matter, which reduces total suspended solids and phytoplankton concentrations, thereby enhancing water clarity and improving overall water quality [4,5,6,7]. Simultaneously, mussels convert ingested organic matter and nutrients into their soft tissues, shells, and biogenic sediments, facilitating nutrient transfer from the water column to the riverbed [8,9]. Therefore, safeguarding freshwater mussel populations is essential for maintaining the integrity of freshwater ecosystems.

The biomass and diversity of freshwater mussels are globally threatened [10]. According to the 2015 IUCN Red List of Threatened Species, 44% of freshwater mussel species are classified as Near Threatened or Threatened. The decline and extinction of these populations are primarily driven by factors such as habitat fragmentation, dam construction, overexploitation, pollution, sediment extraction and dredging, interactions with invasive species, climate change, loss of host fish species, and parasitic infections [10,11,12]. Current research has predominantly focused on the impacts of conventional pollutants, including heavy metals [13], eutrophication [14], and organic contaminants [15]. Recently, microplastics (MPs, 1–5 mm) and nanoplastics (NPs, 1–1000 nm) have been studied extensively, but there remains a paucity of knowledge on nanoplastic effects with various biological endpoints [16]. Bivalves are particularly vulnerable to ingesting plastic particles due to their filter-feeding mechanisms, which passively capture these particles from the water [17].

Studies have shown that MPs can have both direct and indirect adverse effects on bivalves, such as physiological impairments, habitat structure alterations, disruption of food sources, and increased persistence of organic pollutants [18]. Although studies on plastics have primarily focused on MPs and marine environments, there has been relatively little research on smaller-sized NPs, especially for freshwater ecosystems [19]. That is despite the potential for NPs to have more acute impacts than MPs. Due to their larger surface area-to-volume ratio, smaller size, and higher aggregation tendency, NPs penetrate tissues more rapidly than MPs and may exhibit greater biological toxicity [19,20]. Studies have demonstrated that NPs can cause various physiological effects in bivalves, including altered lifecycles, oxidative stress, histological changes, neurotoxicity, immunotoxicity, genotoxicity, and proteomic profiles [17,21,22]. The biotoxicity of NPs in marine mussels has been extensively studied [23,24,25]. For instance, in vitro exposure to 0.08 and 10 μg·L^−1^ polystyrene nanoplastic (PS-NPs, 200 nm) has been shown to cause hemocyte mortality and DNA damage in *Mytilus edulis* [26], while 20 μg·L^−1^ PS-NPs (139.5 nm) significantly disrupted the intestinal microbiota of *Ruditapes philippinarum* [22]. Freshwater mussel habitats, which are often located in hotspots of human activity and which exhibit heightened diversity, are more susceptible to anthropogenic impacts than marine mussel habitats but remain understudied [27].

Considering the essential role of freshwater mussels in ecosystem function and their threatened status, investigating the effects of NPs on these species is crucial since NPs are globally widespread and are detected in various water sources, including rivers, lakes, and freshwater sediments. For instance, Materić et al. [28] detected mean NP concentrations of 51 μg·L^−1^ in Siberian and 563 μg·L^−1^ in Swedish freshwaters. Similarly, Junaid et al. [29] reported PS-NP concentrations in the Pearl River of 29.6–1504.4 μg·L^−1^. In the Chinese Lake Taihu, combined concentrations of MPs and NPs were observed in sediments, measuring 11.0–234.6 items/kg dry weight (dw) [30]. However, NP particle counts may exceed MPs by up to 10^14^ times [31]. PS-NPs are commonly found in freshwater systems, with 50 nm PS-NPs showing higher cytotoxicity than larger particles in human gastric cells [32,33]. Most studies have used MP/NP concentrations of 100–1000 mg·L^−1^ (exceeding environmental concentrations) and focused solely on adult or juvenile stages of mussels [34,35]. However, juveniles may be more sensitive to environmental stressors, such as acute cadmium exposure, hypoxia, high temperature, and acidity, compared to adults [36,37]. Therefore, studying the effects of varying NP concentrations on freshwater mussels across different growth stages, as well as with concentrations that are more environmentally relevant, could provide a crucial foundation for evaluating the ecological risks of NPs to these organisms.

The gut microbiota is closely linked to the host’s nutritional metabolism and immune regulation, playing a crucial role in protecting against pathogens, producing beneficial secondary metabolites, and aiding in the digestion of complex nutrients [38]. The gut microbiota has been shown to be highly sensitive to environmental pollutants, and its disruption can negatively impact marine host health status [39]. In particular, the intestinal gland is essential for digestion, absorption, and immune function in mussels, serving as a primary target for exogenous substances [40]. Most current research suggests that NPs and MPs have several effects on the intestine of marine organisms (such as fish, mussels, and zooplankton): inflammation, reduced mucus secretion, damage to the intestinal epithelial mucosa, and ecological dysbiosis [41]. However, the impact of NPs on the intestinal microbiota of freshwater mussel (Unionoida) species remains unexplored.

This study conducted a mesocosm experiment to assess the effects of realistic and elevated ambient NP concentrations on freshwater mussels at various growth stages, using *Cristaria plicata* as a model species commonly found in East Asia [42,43]. To characterize the toxic mechanism of NPs on *C. plicata*, we investigated the effects on intestinal tissue, physiological and biochemical indicators such as MDA, SOD, ammonia excretion rate (AER), respiration rate (RR), total protein (TP), and the intestinal microbiota. We hypothesize that: (1) Exposure to 50 nm PS-NPs will significantly impact physiological parameters and gut microbiota composition in *C. plicata*, with toxicity being dose-dependent; (2) juvenile mussels will show greater sensitivity to PS-NPs (50 nm) exposure than adults, demonstrating more significant physiological and microbiota changes. Overall, this study builds an understanding of the impacts of PS-NPs on various physiological endpoints while assessing dose, ontogenic, and temporal context dependencies.

## 2. Materials and Methods

### 2.1. Materials of PS-NPs

Polystyrene monodisperse microspheres suspension (PS-NPs; *w*/*v*: 2.5%; diameter: ~50 nm) were purchased from Jiangsu Zhichuan Technology Co., Ltd. (Nantong, China). The stock solution is a mixture of polystyrene and pure water.

*Cristaria plicata* were sampled through manual random collection from Honghu Lake, near the Yangtze River in China. Adult and juvenile mussels were separately acclimated in glass aquaria (volume: 60 L, 50 cm × 40 cm × 30 cm), using artificial river water and freshwater river sand (0–2 mm), with eight individuals placed in each tank (26 glass tanks in total) for both age groups. During this period, mussels were supplemented daily with 0.5% dry *Spirulina* powder based on mussel biomass [44], and low-vigor individuals (mussels exhibiting a delayed or impaired shell-closing response to forceps touch) were removed. The acclimation and experimental water consisted of artificial river water, maintained at a pH of 7.57 ± 0.25, a temperature of 15 ± 1.15 °C, and a dissolved oxygen level of 7.64 ± 0.23 mg/L, with an acclimation period of 3 weeks.

### 2.2. Experimental Design

Hermaphroditic *C. plicata* capable of sexual transformation underwent sex differentiation at 5 cm body length and reached sexual maturity at ~10 cm [45]. Adult mussels (10–13 cm) and juveniles (4–7 cm) were separately cultured in glass tanks after acclimatization for the experiment. Four treatments were established, corresponding to growth stages—juveniles and adults. Juvenile mussels were exposed to 2 μg·L^−1^ (Juvenile mussel-Low concentration, J-L), 20 μg·L^−1^ (Juvenile mussel-Medium concentration, J-M), and 200 μg·L^−1^ PS-NPs (Juvenile mussel-High concentration, J-H), and a control group without PS-NPs (Juvenile mussel-control, J-CK) was established. Adult mussels received the same treatments and were labeled A-L, A-M, A-H, and A-CK. Each treatment included three replicate 60 L glass aquaria (50 cm × 40 cm × 30 cm) with eight individuals per tank (*n* = 24 per treatment). The experimental culture conditions were similar to the acclimation period. No mortality or abnormalities were observed throughout the entire duration.

A diluted solution of uniformly distributed monodisperse PS-NPs microspheres in purified water was achieved through ultrasound (80 kHz) exposure for 5 min. This solution was used as culture media and was maintained in glass containers at 18 ± 0.55 °C with a 12:12 h light–dark cycle. Sand was cleaned and replaced every three days, with 0.5% biomass *Spirulina* supplement provided daily at 9:00–10:00 AM [44].

### 2.3. Characterization of PS-NPs

The size and morphology of PS-NPs in the stock solution were examined using a Transmission Electron Microscope (TEM, JEOL, JEM-F2100, Tokyo, Japan), and the size range was found to be 50 ± 0.3 nm (Figure S1). Fourier Transform Infrared (FTIR) spectroscopy (Nicolet Nexus 470, Thermo Fisher Scientific, Durham, NH, USA) was used to gather infrared spectral data from NP material samples used in this experiment and confirmed that the characteristic absorption peaks correspond to the structural features of PS-NPs (Figure S2).

### 2.4. Ultrastructural Morphology Analysis of Intestinal Tract

On day 45 of exposure, a mussel was randomly chosen from each tank (*n* = 3 per treatment) for intestinal dissection to examine epithelial tissue changes. The intestinal tissue samples were immersed in 2.5% glutaraldehyde buffer for 4 h, then rinsed with PBS (0.1 M, pH 7.0) for 15 min. This process was repeated once more, with a total of three rinses [22]. Subsequently, the samples underwent dehydration using a graded ethanol series (50%, 70%, 80%, 90%, and 95%) for successive intervals, followed by a final 20-min immersion in 100% ethanol. Ultrastructural morphology analysis of the intestinal tract was conducted using a scanning electron microscope (SEM; CLARA, TESCAN, Brno, Czech Republic) with three samples per condition [46].

### 2.5. Physiological and Biochemical Parameters

#### 2.5.1. Oxidative Stress Markers and Total Protein Content

One mussel was randomly selected from each tank (*n* = 3 per treatment) to determine superoxide dismutase (SOD) and malondialdehyde (MDA) levels at 15 (T15), 30 (T30), and 45 (T45) days after exposure. Intestinal tissue was dissected, homogenized in 0.9% NaCl (pH: 7.4) at a ratio of 1:9, and centrifuged at 4000 rpm for 10 min. The levels of SOD, MDA, and total protein (TP) content were measured with 50 µL supernatants using kits from the Nanjing Jiancheng Bioengineering Institute [47]. The SOD assay kit, based on the xanthine oxidase method, detected all SOD isozymes (Cu/Zn-, Mn-, Fe-SOD) without cross-reactivity with CAT or GSH-Px, with a detection range of 4.1–100.1 U·mg^−1^ protein in *C. plicata*. The MDA detection kit employed a thiobarbituric acid (TBA) colorimetric method (range: 0.4–92.6 nmol·mg^−1^ protein), with interference from other aldehydes minimized by distinct absorption spectra (HNE-TBA at 450 nm vs. MDA-TBA at 532 nm). The TP assay kit was based on the Coomassie Brilliant Blue (Bradford) method, with a detection range of 0.2–1.3 g·L^−1^, approximately four times higher than that of the Lowry method. This assay demonstrates superior resistance to common interfering substances, including K^+^, Na^+^, Mg^2+^, Tris buffer, sucrose, and EDTA, which were known to affect the Lowry method.

#### 2.5.2. Physiological Metabolic Indexes at the Individual Level

Mussel respiratory rate (RR, mg O_2_·L^−1^) was assessed by measuring dissolved oxygen (DO) (mg O_2_·L^−1^) in water using an optical electrode (YSI, EcoSense ODO200, Yellow Springs, OH, USA). One mussel per condition (three replicates) was placed in a 1 L beaker with artificial river water, and DO was measured again after three hours with the electrode. Control beakers without mussels were used for comparison. RR was calculated as per [48]:

RR = (C_0_ − C_t_ − δC) × V/N∙t (1)

where RR is the respiration rate (mg O_2_·h^−1^), V is the volume of river water in the container (L), C_0_ is the initial DO (mg O_2_·h^−1^), C_t_ is the final DO at time t (mg O_2_·h^−1^), δC is the change in DO (mg O_2_·h^−1^) using a control beaker without mussels, N is the number of mussels in the tanks, and t is the time elapsed (h).

The ammonia excretion rate (AER) was measured by monitoring the change in ammonia concentration in water samples. A 25 mL sample was filtered using fiberglass filter paper GF/F (1825-047, Whatman, Kent, UK) to remove impurities. The ammonia nitrogen concentration was determined using Nessler’s reagent spectrophotometry. The AER was calculated by subtracting the ammonia concentration in containers without mussels from that with mussels, as per the following formula [48]:

AER = (C_t_ − C_0_ − δC) × V/N∙t (2)

where AER is the ammonia excretion rate (mg NH_3_-N·h^−1^), V is the volume of test seawater in the container (L), C_0_ is the initial ammonia concentration (mg NH_3_-N·h^−1^), C_t_ is the ammonia concentration at time t (mg NH_3_-N·h^−1^), δC is the change in ammonia concentration (mg NH_3_-N·h^−1^) using a control beaker without mussels, N is the number of mussels in the tanks, and t is the time elapsed (h).

### 2.6. Gut Microbiota DNA Extraction and High-Throughput Sequencing

The intestinal contents of three mussels were randomly selected from each treatment group and divided into three samples. Their content was scraped into centrifuge tubes and stored at −80 °C for subsequent analysis. DNA extraction was performed using the E.Z.N.A. Stool DNA Kit (Omega Bio-tek, Inc., Norcross, GA, USA), and the extracted DNA quality and concentration were assessed using Nanodrop 2000 (ThermoFisher Scientific, Inc., Wilmington, DE, USA). The samples underwent PCR amplification and high-throughput sequencing on the Applied Biosystems and Illumina MiSeq platforms. The sequencing data were filtered and assembled using Pear (v0.9.6) to improve data quality. Subsequently, Vsearch (v2.7.1) was used to remove sequences < 230 bp and to identify and remove chimeric sequences using the Gold Database and the uchime algorithm, ensuring high data quality for analysis [49,50,51]. The Vsearch (v2.7.1) software, utilizing the uparse algorithm, was used to cluster high-quality sequences into operational taxonomic units (OTUs) with a 97% sequence similarity threshold [50,52]. Subsequently, OTUs were normalized at the phylum level, and diversity analysis was conducted using the relative abundance of OTUs in each experimental group.

### 2.7. Statistical Analysis

Physiological metabolism and enzymatic activity data (MDA and SOD activities) were analyzed using SPSS software (Stastics22, IBM, Armont, NY, USA). Levene’s test was conducted to test the homogeneity of variances in time series data (MDA, SOD, RR, AER, TP). A three-way (age, concentration, duration) ANOVA (analysis of variance) was performed to evaluate oxidative stress biomarkers (MDA, SOD) and physiological parameters (RR, AER, TP) in both age groups. Within each time point, one-way ANOVA identified intergroup differences. Tukey’s test was applied post hoc to evaluate if the differences were significant. Data normality (MDA, SOD, RR, AER, TP) was verified using the Shapiro–Wilk test. Non-normal data were analyzed with the Kruskal–Wallis test, followed by Dunn’s post hoc test where applicable. All statistical analyses were conducted at *p* < 0.05.

α-diversity indices and β-diversity Bray–Curtis’s distance matrices were calculated using QIIME (v1.7.0), and graphs were generated using R software (v2.15.3) [53,54]. The α-diversity in this study was characterized using the Chao1 index, with β-diversity visualized via principal coordinate analysis (PCoA). A Venn diagram was used to statistically compare the composition of OTUs between groups. Furthermore, Welch’s t-test analysis was performed using the STAMP software to assess the relative abundance differences of microbial phyla between different groups [55].

Graphs for physiological metabolism and enzymatic activity results were generated using the Origin2022 software (Origin Lab Corp, Northampton, MA, USA), while intestinal microbiota data were visualized using R (v2.15.3) software.

## 3. Results

### 3.1. Effects of PS-NPs on Intestinal Tissue

**Figure 1:**
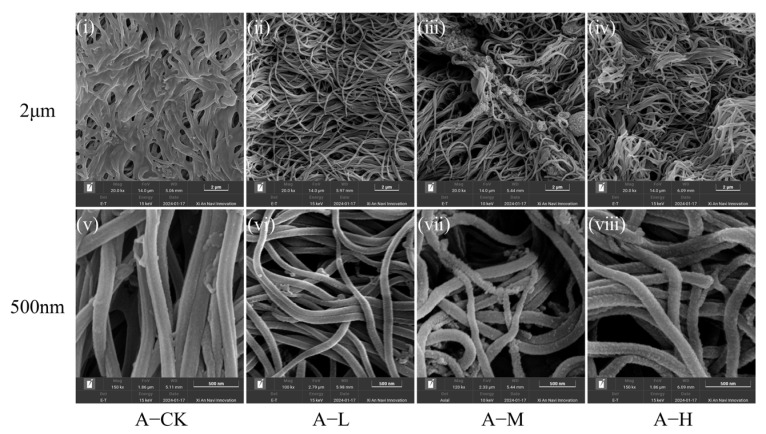
Scanning electron microscope (SEM) of the intestinal gland mucosa in adult *C. plicata* under different concentrations of NP exposure (Scale: 500 nm/2 μm). (**i**–**iv**) SEM images of adult *C. plicata* intestinal gland mucosa exposed to varying NP concentrations (2 μm scale). (**v**–**viii**) Corresponding SEM images at 500 nm scale. Adult mussel exposed to 2 μg·L^−1^ (Adult mussel-Low concentration, A-L), 20 μg·L^−1^ (Adult mussel-Medium concentration, A-M), and 200 μg·L^−1^ PS-NPs (Adult mussel-High concentration, A-H) and a control group without PS-NPs (Adult mussel-Control, A-CK).

From SEM under a 2 μm scale, the intestinal gland mucosa of adult mussels in the A-CK and A-L groups was densely packed with intact morphology cilia (Figure 1i,ii). The A-CK group filled with mucus (Figure 1i). However, the A-M and A-H groups exhibited fewer tangled cilia accumulated with food vacuoles (Figure 1iii,iv). Under a 500 nm scale, intact ciliary structures were observed in the A-CK and A-L, but rough surfaces with damaged structures and granular deposits were present in the A-M (Figure 1vii) and A-H, which had a substantial surface accumulation of granular material (Figure 1viii).

**Figure 2:**
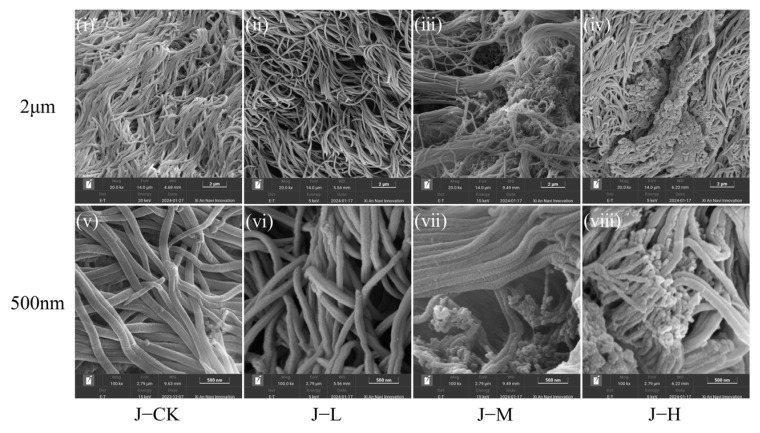
Scanning electron microscope (SEM) of the intestinal gland mucosa in juvenile *C. plicata* under different concentrations of NP exposure (Scale: 500 nm/2 μm). (**i**–**iv**) SEM images of juvenile *C. plicata* intestinal gland mucosa exposed to varying NP concentrations (2 μm scale). (**v**–**viii**) Corresponding SEM images at 500 nm scale. Juvenile mussels exposed to 2 μg·L^−1^ (Juvenile mussel-Low concentration, J-L), 20 μg·L^−1^ (Juvenile mussel-Medium concentration, J-M), and 200 μg·L^−1^ PS-NPs (Juvenile mussel-High concentration, J-H) and a control group without PS-NPs (Juvenile mussel-control, J-CK).

In juvenile mussels, the cilia in the J-CK and J-L were closely arranged, but the epithelial surface lacked digestive fluids (Figure 2i,ii). The J-M had damaged cilia with a disrupted distribution, while the J-H exhibited extensive food vacuole aggregation (Figure 2iii,iv). Under a 500 nm scale, the intestinal cilia of juvenile mussels in J-CK and J-L were intact and smooth, contrasting with the rough, broken, and particle-substance-filled cilia in J-M and J-H (Figure 2). Juveniles in the J-M/J-H exhibited more severe ciliary breakage and damage compared to adults (Figure 2vii,viii).

Consequently, low-concentration PS-NPs preserved cilia integrity in both adult and juvenile mussel intestinal epithelium, while medium to high concentrations led to cilia disruption and damage.

### 3.2. Effects of PS-NPs on Oxidative Stress Response and Total Protein

**Figure 3:**
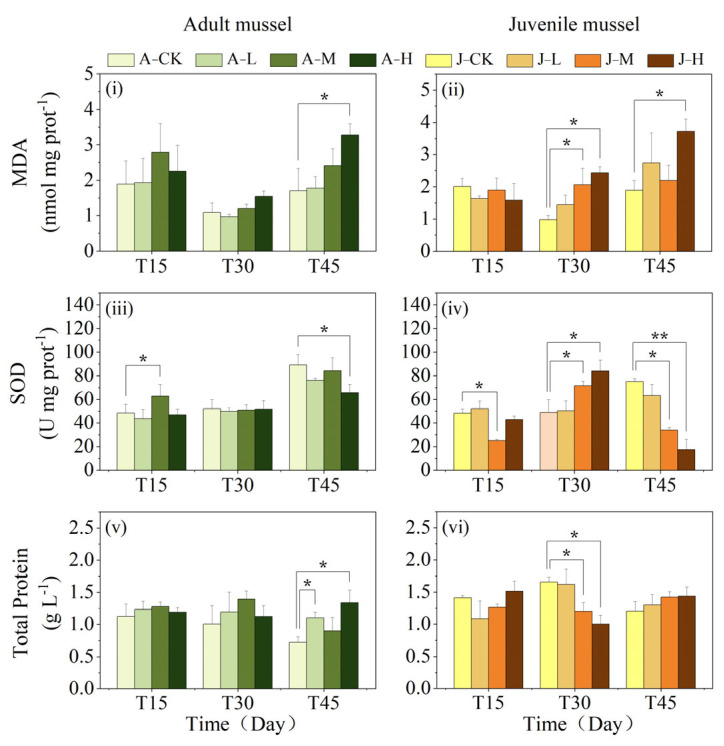
PS-NPs effect on malondialdehyde (MDA) level, superoxide dismutase (SOD) activities, and total protein (TP) content in *C. plicata*. Adult mussel exposed to 2 μg·L^−1^ (Adult mussel-Low concentration, A-L), 20 μg·L^−1^ (Adult mussel-Medium concentration, A-M), and 200 μg·L^−1^ PS-NPs (Adult mussel-High concentration, A-H) and a control group without PS-NPs (Adult mussel-Control, A-CK). Juvenile mussels exposed to 2 μg·L^−1^ (Juvenile mussel-Low concentration, J-L), 20 μg·L^−1^ (Juvenile mussel-Medium concentration, J-M), and 200 μg·L^−1^ PS-NPs (Juvenile mussel-High concentration, J-H) and a control group without PS-NPs (Juvenile mussel-control, J-CK). (**i**,**ii**) MDA, (**iii**,**iv**) SOD, and (**v**,**vi**) total protein (TP) changes in adults and juveniles. The asterisk indicates a significant difference between the exposed group and the control group at a time point (* *p* < 0.05; ** *p* < 0.01).

The MDA activity in freshwater mussels was significantly influenced by duration, concentration, and the interactions between age and duration, as well as between concentration and duration (Table 1). The interaction between age and exposure duration was reflected in the significant changes in MDA activity in juveniles compared to adults at shorter exposure times (Figure 3i,ii). The interaction between concentration and duration was reflected in the MDA activity in freshwater mussels at higher concentrations, which showed significant changes in both age groups within a shorter time, as opposed to the effects observed at lower concentrations (Figure 3i,ii).

The individual effects of exposure concentration and duration on adult and juvenile mussels were as follows. In adult mussels, a significant increase in MDA activity was only observed in the high-concentration group after 45-day exposure (*p* < 0.05), with no significant differences detected among any exposure groups and the control at either 15 or 30 days (Figure 3i). In juvenile mussels, there were no significant differences in MDA activity between exposed groups and the control (J-CK) at 15 days (Figure 3ii). At 30 days, MDA activity in the J-M and J-H groups was significantly higher than the J-CK group (*p* < 0.05), while the J-L group did not differ significantly from the J-CK group. At 45 days, MDA activity in the J-H group was significantly higher than in the J-CK group (*p* < 0.05), with no significant differences in the other exposed groups compared to the J-CK group.

The SOD activity in freshwater mussels was significantly influenced by age, duration, concentration, as well as their pairwise and three-way interactions (Table 1). In detail, age interacts with PS-NP concentration and exposure duration, as is reflected in the significant changes of SOD observed in juvenile mussels compared to adult mussels at shorter exposure times or lower concentrations (Figure 3iii,iv). The interaction of exposure concentration and duration significantly affected SOD activity in both age groups (Figure 3iii,iv). In adults, SOD activity changed significantly in the high-concentration group first, while in juveniles, significant changes occurred more rapidly at medium and high concentrations compared to low concentrations. Additionally, the three-way interaction between PS-NP exposure concentration, exposure duration, and age reflected distinct differences between adult and juvenile responses post-exposure, which varied with PS-NP exposure time and concentration (Figure 3iii,iv). For example, at high concentrations, juvenile SOD activity was less affected initially but decreased significantly with prolonged exposure.

In adults, the A-M group had significantly higher SOD activity than the A-CK and J-M groups after 15 days of exposure (*p* < 0.05; Figure 3iii). By 30 days, there were no significant changes in SOD activity among exposed adult groups compared to the A-CK group, but the medium and high exposure groups had significantly lower SOD activity than juveniles (*p* < 0.05). On 45 days, the A-H group had significantly lower SOD activity than the A-CK group (*p* < 0.05), and all exposed adults had higher SOD activity than juveniles (*p* < 0.05). In juveniles, the J-M group had significantly lower SOD activity than the J-CK group at 15 days (*p* < 0.05), with no significant differences in other exposed groups (Figure 3iv). By 30 days, the J-M and J-H groups had significantly higher SOD activity than the J-CK group (*p* < 0.05), while the J-L group did not differ. At 45 days, SOD activity in exposed groups decreased in a dose-dependent manner, with the J-M and J-H groups showing significantly lower activity than the J-CK group (*p* < 0.05) and the J-L group showing no difference.

The mussel total protein (TP) content was significantly affected by age, age–concentration, concentration–duration, and age–duration–concentration interactions (Table 1). At 30 days of exposure, the medium-concentration groups’ adult mussels had significantly higher TP content than juvenile ones, with no significant difference in the other groups, but with the opposite shown at 45 days (*p* < 0.05; Figure 3v,vi). For adults, TP content in the A-L and A-H groups significantly increased compared to the A-CK group by 45 days (*p* < 0.05), and no significant difference was found in A-M (Figure 3v). There were no significant differences among the 15 and 30 days of exposure groups. For juveniles, at 15 days, there were no significant differences between the exposed groups and J-CK. On 30 days, the TP content in J-M and J-H groups was significantly lower than in J-CK (*p* < 0.05). All exposed groups showed no significant TP content differences compared to controls at 45 days (Figure 3vi). Moreover, the age–concentration interaction led to significantly reduced TP in juveniles at medium–high concentrations but increased TP in adults at medium concentrations after 30-day exposure. The interaction of concentration and PS-NP exposure duration showed that juvenile mussel TP changed earlier at the high and medium concentrations compared to the low-concentration group, whereas significant changes occurred in adult mussels at both low and high concentrations before medium concentrations.

### 3.3. Effects of PS-NPs on Physiological Metabolism at the Individual Level

**Figure 4:**
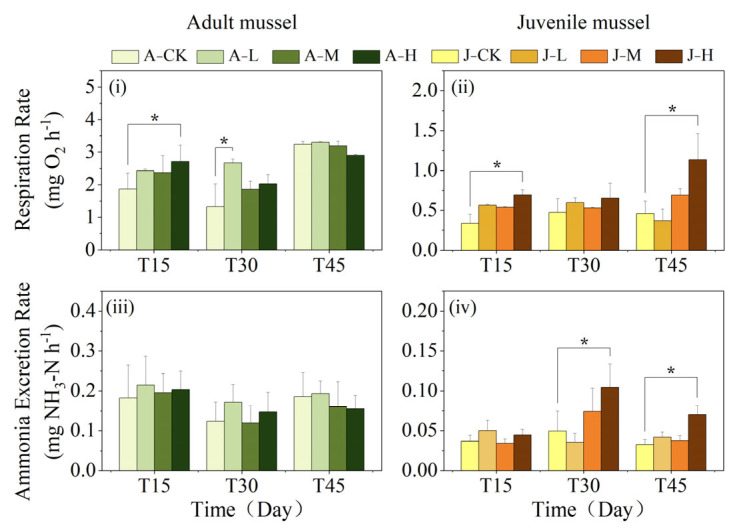
Effect of PS-NPs on the respiratory rate (RR) (**i**,**ii**) and ammonia excretion rate (AER) (**iii**,**iv**) of adult and juvenile mussels. Adult mussel exposed to 2 μg·L^−1^ (Adult mussel-Low concentration, A-L), 20 μg·L^−1^ (Adult mussel-Medium concentration, A-M), and 200 μg·L^−1^ PS-NPs (Adult mussel-High concentration, A-H) and a control group without PS-NPs (Adult mussel-Control, A-CK). Juvenile mussels exposed to 2 μg·L^−1^ (Juvenile mussel-Low concentration, J-L), 20 μg·L^−1^ (Juvenile mussel-Medium concentration, J-M), and 200 μg·L^−1^ PS-NPs (Juvenile mussel-High concentration, J-H) and a control group without PS-NPs (Juvenile mussel-control, J-CK). The asterisk indicates a significant difference between the exposed group and the control group at a time point (* *p* < 0.05).

The mussel respiratory rate (RR) was significantly influenced by age, concentration, time, and the interaction between age and duration (Table 2). Adult mussels had significantly higher RR than juveniles at all exposure times (Table 2; Figure 4i,ii). In adults, the A-H group had a significantly higher RR than the A-CK group at 15 days (*p* < 0.05), while other groups did not differ from A-CK (Figure 4i). At 30 days, the A-L group had a significantly higher RR than A-CK (*p* < 0.05). By 45 days, no significant differences were found between the exposure group and the control. In juveniles, the J-H group had a significantly higher RR than the J-CK group at 15 and 45 days (*p* < 0.05), but no significant differences between the exposure group and control at 30 days (Figure 4ii).

The ammonia excretion rate (AER) of mussels was both interactively influenced by age and exposure duration and independently affected by age alone (Table 2). Adults’ AER was always significantly higher than juveniles (*p* < 0.05; Figure 4iii,iv). Adults among exposure groups all showed no significant difference from A-CK across sampling times (Figure 4iii). However, juveniles had significantly higher AER in the J-H group than in the J-CK group after 30 and 45 exposure days (*p* < 0.05).

### 3.4. Effects of PS-NPs on Gut Microbiota

#### 3.4.1. Effects of PS-NPs on Gut Microbiota Diversity

**Figure 5:**
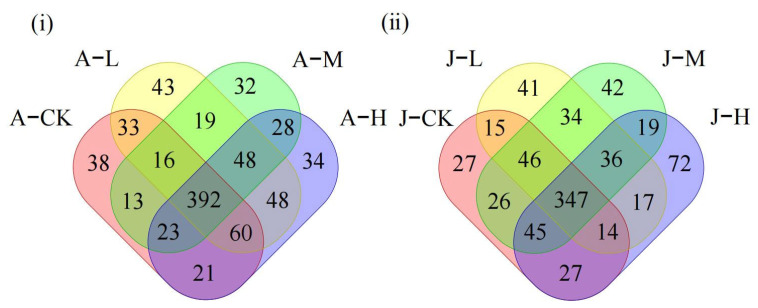
Venn diagrams of the *C. plicata* intestinal microbiota exposed to different concentrations of PS-NPs. (**i**) The shared operational taxonomic units (OTUs) count between the control and exposed adult mussel groups; (**ii**) The shared OTUs count between the control and exposed juvenile mussel groups. Adult mussels exposed to 2 μg·L^−1^ (Adult mussel-Low concentration, A-L), 20 μg·L^−1^ (Adult mussel-Medium concentration, A-M), and 200 μg·L^−1^ PS-NPs (Adult mussel-High concentration, A-H) and a control group without PS-NPs (Adult mussel-Control, A-CK). Juvenile mussels exposed to 2 μg·L^−1^ (Juvenile mussel-Low concentration, J-L), 20 μg·L^−1^ (Juvenile mussel-Medium concentration, J-M), and 200 μg·L^−1^ PS-NPs (Juvenile mussel-High concentration, J-H) and a control group without PS-NPs (Juvenile mussel-control, J-CK).

The 16S rRNA sequencing of the adult *C. plicata* intestinal microbiota revealed 596, 659, 571, and 654 operational taxonomic units (OTUs) across different PS-NP exposure concentrations (Figure 5i). The A-L, A-M, and A-H groups shared 33, 13, and 21 OTUs with the control, respectively, with the A-L group having the most shared OTUs and the A-M group the least (Figure 5i). In juveniles, 547, 550, 595, and 577 OTUs were detected (Figure 5ii), and the J-L, J-M, and J-H groups shared 15, 26, and 27 OTUs with the control, respectively. The J-H group exhibited the highest, while the J-L group shared the fewest number of unique OTUs with the J-CK group (Figure 5ii). The decline in the shared OTU count between adult mussels in exposed groups and the A-CK was greater than that between juvenile mussels in exposed groups and the J-CK (Figure 5 iii,iv).

**Figure 6:**
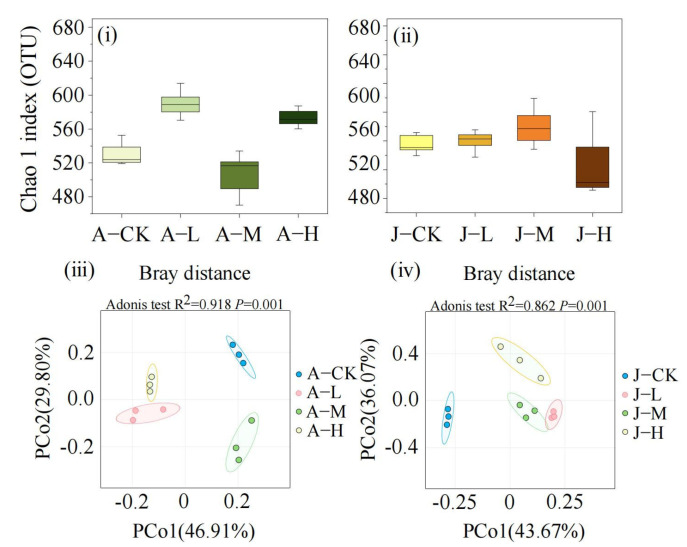
α- and β-diversity analysis of the *C. plicata* intestinal microbial communities. (**i**,**ii**) The Chao1 index; (**iii**,**iv**) The PCoA analysis of the adult and juvenile mussel intestinal microbial communities, respectively, 45 days post-exposure to PS-NPs. CK groups are the totals shared for the bar plots. Adult mussel exposed to 2 μg·L^−1^ (Adult mussel-Low concentration, A-L), 20 μg·L^−1^ (Adult mussel-Medium concentration, A-M), and 200 μg·L^−1^ PS-NPs (Adult mussel-High concentration, A-H) and a control group without PS-NPs (Adult mussel-Control, A-CK). Juvenile mussels exposed to 2 μg·L^−1^ (Juvenile mussel-Low concentration, J-L), 20 μg·L^−1^ (Juvenile mussel-Medium concentration, J-M), and 200 μg·L^−1^ PS-NPs (Juvenile mussel-High concentration, J-H) and a control group without PS-NPs (Juvenile mussel-control, J-CK).

In adult mussels, α-diversity analysis indicated that the A-L and A-H groups exhibited significantly higher microbial diversity (Chao1 index) than the control (*p* < 0.05), whereas the A-M group showed no significant difference (Figure 6i). For juvenile mussels, the J-L and J-M groups displayed microbial diversity similar to the control (J-CK), while the J-H group had reduced diversity (Figure 6ii). β-diversity was visualized using PCoA based on Bray-Curtis distances, and significant differences among groups were confirmed by Adonis test (*p* < 0.05). In adult mussels, the principal components PCo1 and PCo2 accounted for 29.8% and 46.91% of the microbial community structure variability, highlighting significant differences between exposure groups and the control (Figure 6iii). In juvenile mussels, the first and second PCo1 and PCo2 axes explained 36.07% and 43.67% of the variation, respectively (Figure 6iv). Confidence ellipses for adult groups did not overlap, indicating distinct β-diversity differences, whereas some overlap was observed between J-L and J-M groups, suggesting less pronounced differences among juvenile mussel exposure groups.

#### 3.4.2. Effects of PS-NPs on Gut Microbiota Composition

**Figure 7:**
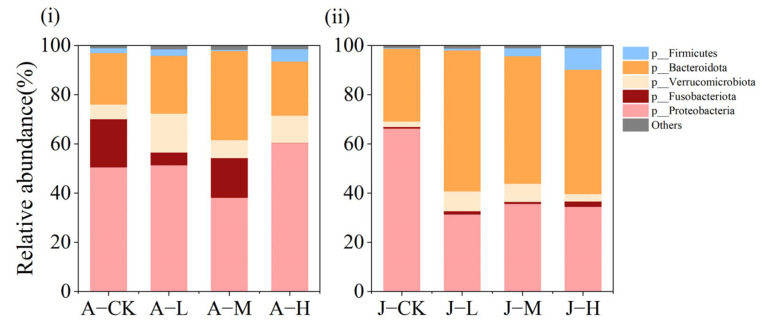
Bar plot of relative abundance of microbial communities at the phylum level in *C. plicata* considering adults (**i**) and juveniles (**ii**). CK groups are the totals shared for the bar plots. Adult mussel exposed to 2 μg·L^−1^ (Adult mussel-Low concentration, A-L), 20 μg·L^−1^ (Adult mussel-Medium concentration, A-M), and 200 μg·L^−1^ PS-NPs (Adult mussel-High concentration, A-H) and a control group without PS-NPs (Adult mussel-Control, A-CK). Juvenile mussels exposed to 2 μg·L^−1^ (Juvenile mussel-Low concentration, J-L), 20 μg·L^−1^ (Juvenile mussel-Medium concentration, J-M), and 200 μg·L^−1^ PS-NPs (Juvenile mussel-High concentration, J-H) and a control group without PS-NPs (Juvenile mussel-control, J-CK).

Figure 7 illustrates the abundance of the five most common bacterial phyla in the intestinal microbiota of *C. plicata*, which are Proteobacteria, Bacteroidota, Verrucomicrobiota, Firmicutes, and Fusobacteriota. In adult mussels from the A-CK, A-L, and A-M groups, as well as in all juvenile groups, the dominant phyla were primarily Proteobacteria, Bacteroidota, Verrucomicrobiota, Firmicutes, and Fusobacteriota. Notably, Fusobacteriota abundance was significantly lower in the A-H group than in other groups of adult mussels.

**Figure 8:**
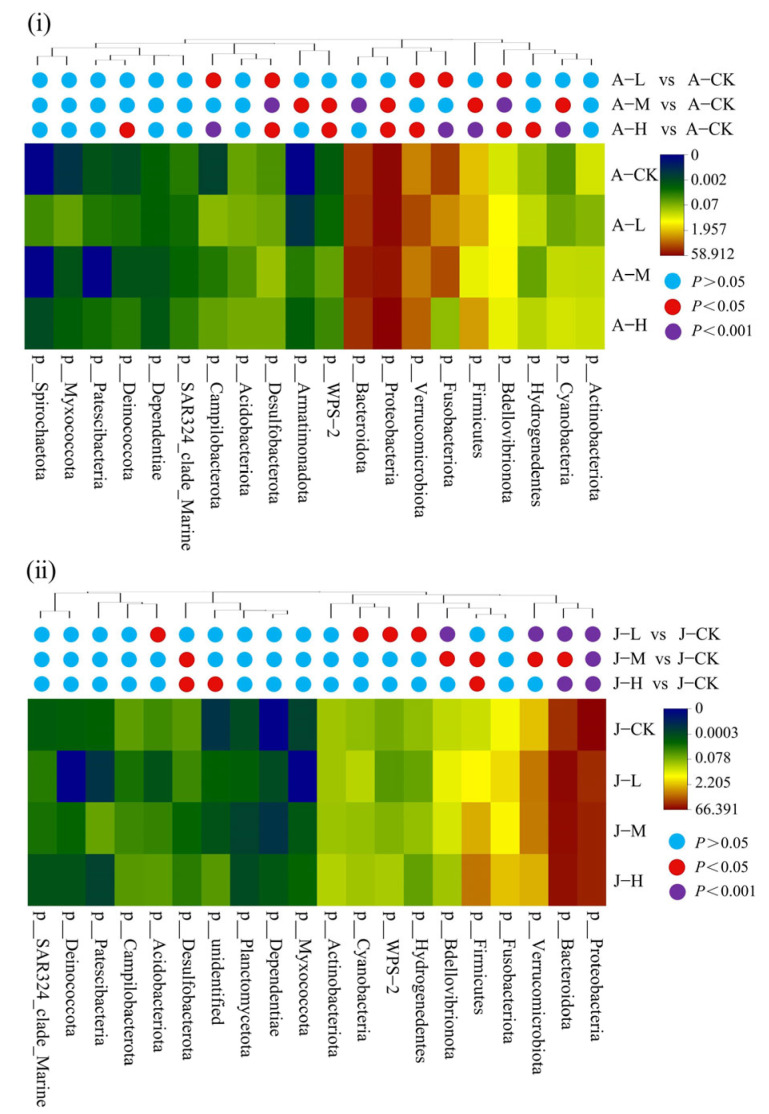
Heatmap of phylum-level differences in the biotic community in *C. plicata* considering adults (**i**) and juveniles (**ii**). CK groups are the totals shared for the bar plots. Adult mussel exposed to 2 μg·L^−1^ (Adult mussel-Low concentration, A-L), 20 μg·L^−1^ (Adult mussel-Medium concentration, A-M), and 200 μg·L^−1^ PS-NPs (Adult mussel-High concentration, A-H) and a control group without PS-NPs (Adult mussel-Control, A-CK). Juvenile mussels exposed to 2 μg·L^−1^ (Juvenile mussel-Low concentration, J-L), 20 μg·L^−1^ (Juvenile mussel-Medium concentration, J-M), and 200 μg·L^−1^ PS-NPs (Juvenile mussel-High concentration, J-H) and a control group without PS-NPs (Juvenile mussel-control, J-CK).

In contrast to juvenile mussels, the number of significantly changed bacterial phyla in the exposure groups of adult mussels increased with concentration (A-L: 5, A-M: 8, A-H: 11; J-L: 8; J-M: 6; J-H: 5; Figure 8). In adults, Welch’s t-test identified significant changes in the relative abundance of 13 bacterial phyla (*p* < 0.05; Figure 8i). Compared to A-CK, Proteobacteria and Firmicutes decreased significantly in A-M (*p* < 0.05), increased significantly in A-H (*p* < 0.05), and remained unchanged in A-L. Bacteroidota in A-M and Verrucomicrobiota in A-L/A-H were significantly increased. Fusobacteriota showed significantly lower abundance in both A-L and A-H compared to the control (*p* < 0.05). In juveniles, significant changes (*p* < 0.05) were observed in the relative abundance of 11 bacterial phyla (Figure 8ii). Proteobacteria abundance decreased significantly in all exposed groups compared to the J-CK, while Bacteroidota increased significantly (*p* < 0.05). Verrucomicrobiota abundance in J-M/J-H also significantly rose (*p* < 0.05). Firmicute abundance increased with PS-NP concentration, with J-M and J-H showing significantly higher levels than the control (*p* < 0.05). Fusobacteriota abundance remained constant across juvenile groups. Overall, adult mussels exhibited a broader spectrum of microbial phyla affected by PS-NP exposure compared to juvenile mussels (Figure 8i,ii).

## 4. Discussion

The study found that adult and juvenile mussels exhibit different physiological and intestinal microbial changes in response to PS-NPs. The three-way ANOVA results indicated that age, PS-NP exposure duration, and concentration were key factors significantly affecting physiological and biochemical parameters in *C. plicata*. Juvenile mussels’ intestinal tissue, SOD, RR, and AER were more significantly affected by PS-NPs, whereas the intestinal microbiota of adult mussels was more influenced by PS-NPs. Compared to the control group, medium and high-concentration exposure groups showed damage to the cilia of the intestinal mucosal epithelium in both adult and juvenile mussels and reduced mucus secretion in adults. Additionally, 45-day exposure to PS-NPs increased the MDA levels and TP content in adult mussels, as well as the MDA levels, RR, and AER in juvenile mussels, decreased the SOD activity in both adult and juvenile mussels, and altered the diversity and composition of key gut microbial phyla. In contrast to juvenile mussels, the number of significantly changed bacterial phyla in the various exposure groups of adult mussels increased with concentration, and the gut microbiota diversity and composition of adult mussels underwent more intense changes. The results indicated that PS-NP exposure may cause damage to the intestinal tissue of mussels and induce oxidative stress responses, disrupting the healthy equilibrium of the gut microbiota, with these responses exhibiting concentration-dependent patterns and age-specific mechanisms.

### 4.1. PS-NP Exposure Injured C. plicata Intestinal Tissue

The intestinal gland of mussels plays a critical role in absorbing exogenous substances, making the intestinal epithelial cells vulnerable to damage from micro-nanoplastics and other pollutants [17,56]. In *C. plicata*, the intestinal mucosa predominantly consists of ciliated columnar epithelial cells [57]. This study suggests that the medium- and high-concentration groups showed damage and injury to the cilia of the intestinal epithelial mucosa and no effect in the low-concentration group, confirming that the severity of ciliary impairment increased progressively with higher PS-NP exposure concentrations. This results primarily from two mechanisms. First, PS-NP-induced lipid peroxidation can lead to epithelial cell membrane rupture, as indicated by the significantly elevated MDA levels observed in the high-concentration group. Second, oxidative byproducts from lipid peroxidation can impair intracellular protein synthesis and induce apoptosis in ciliated epithelial cells. Previous studies have associated epithelial injury with cilia aggregation, shedding, breakage, and hemolytic cell infiltration [47,58,59]. Similar to our results, Hariharan et al. [60] found that 30 days of exposure to weathered polyethylene microplastics (wPE-MPs) leads to ciliary loss and damage in the intestinal epithelium of *Perna viridis*. Furthermore, this study observed food vacuole accumulation in the medium and high-exposure groups of mussels, which may result from NPs targeting enterocytes in the intestinal glands responsible for digestion [61] and then reducing digestion. This attack may disturb lysosome stability and mitochondrial function in epithelial cells, triggering apoptosis [24,62]. The higher prevalence of fractured cilia in juvenile mussels compared to adults suggests that PS-NP exposure caused a more pronounced impairment of intestinal structural integrity in juvenile mussels, likely due to their underdeveloped physiology and immune system, which makes them more susceptible to NP effects. Whether the effects on juveniles carry over to adult stages remains a question for future research.

This study observed a reduction in intestinal mucus production in adult mussels exposed to NPs. Mucus serves as a protective barrier, preventing harmful substances from entering and maintaining intestinal microbiota balance. It also contains proteases that assist in nutrient absorption [63]. The reduction in mucus may result from NPs altering the habitat and structure of intestinal microbiota, causing specific bacterial aggregations that compromise the intestinal barrier. Additionally, NPs might increase the sheer force of intestinal peristalsis, intensifying physical damage to the mucus and promoting its degradation [59]. These findings align with studies that observed reduced mucus secretion in the Chinese mussels (*Corbicula fluminea*) exposed to 100 and 1000 μg/L PS-MPs and PS-NPs [64]. The absence of mucus in juvenile mussels might be attributed to their immature developmental stage.

This study highlights that PS-NPs significantly impair the intestinal health of *C. plicata*, with juveniles experiencing more severe damage than adults. Since juveniles are essential for population replenishment, their susceptibility to PS-NPs could hinder the species’ overall growth and reproduction. PS-NPs primarily affect intestinal barrier function and nutrient absorption by damaging cilia and reducing mucus secretion. This impairment may reduce the mussels’ capacity to filter algae and pollutants, thereby affecting the stability and resilience of aquatic ecosystems.

### 4.2. Exposure to PS-NPs Induced C. plicata’s Oxidative Stress Responses

Oxidative stress is a prevalent physiological response to NP exposure in mussels [35,65]. MDA, a product of reactive oxygen species (ROS) attacking cell membrane phospholipids, serves as a marker for lipid peroxidation severity and is associated with cellular and tissue damage [22,66]. In this study, after 30 days, J-H juveniles exhibited significantly elevated MDA levels versus J-CK, with no differences in adults across treatments. This demonstrates stage-specific oxidative stress in juveniles, while in adults, this remained below toxicity thresholds. On 45 days, only the high-concentration group exhibited significantly elevated MDA levels compared to A-CK/J-CK, which may be attributed to cumulative PS-NPs exceeding adult and juvenile detoxification thresholds, indicating prolonged exposure-induced oxidative stress in both juvenile and adult mussels. Intestinal cilia damage increases permeability, facilitating the entry of NPs and harmful bacteria, thereby triggering a stronger stress response. These findings align with previous research indicating that NPs induce excessive ROS production in mussels, leading to severe lipid peroxidation and elevated MDA levels [20].

Enzymatic activity changes often reflect oxidative damage, impaired redox balance, and antioxidant system disruption [20,67,68]. SOD, a key enzyme against ROS, converts O^2−^ to H_2_O_2_ and O_2_, mitigating cell membrane lipid peroxidation [69]. However, the antioxidant defense system can be overwhelmed by chronic high-pollutant exposure, leading to intracellular antioxidant system overload [20,70]. After 30 days, SOD activity in the J-M and J-H juvenile groups significantly exceeded that of J-CK, implying ROS accumulation from PS-NPs activated their antioxidant defense. This aligns with the findings of Zhou et al. [22], which reported SOD activity in *Ruditapes philippinarum* with increasing PS-Pd NP concentration, with more significant ROS accumulation in the high-concentration group. The SOD activity in adults did not show significant changes across treatments, suggesting that their higher stress tolerance thresholds were not exceeded. On 45 days of exposure, SOD activity in adult mussels (A-H) and juvenile mussels (J-M and J-H) was significantly reduced compared to controls. This reduction is attributed to the PS-NP concentration or exposure duration exceeding thresholds, which suppresses antioxidant gene expression, depletes the defense system, and impairs mussels’ ability to regulate SOD activity. The above results confirmed that exposure to 50 nm PS-NPs induces dose-dependent differences in SOD and MDA levels in *C. plicata*.

In response to MP and NP stressors, organisms often reallocate energy and adjust metabolic processes. They may increase protein turnover to meet the energy demands of stress protein production and activate protein degradation pathways [71,72]. In this study, the total protein (TP) content in A-L and A-H groups was significantly higher than the A-CK group after 45 days with NP exposure, likely due to excessive SOD (not limited to SOD) synthesis, with protein synthesis outpacing decomposition. Similarly, Yedier et al. [73] observed that TP content significantly increased in *Cyprinus carpio* juveniles exposed to polypropylene (PP) MPs for seven days. Conversely, the TP content in juvenile mussels decreased after 30 days and remained unchanged after 45 days, possibly due to more severe intestinal damage that reduced nutrient absorption, including amino acids (Figure 1 and Figure 2). This could have affected the juvenile rate of protein synthesis at 30 days. Additionally, juveniles also require more energy from protein breakdown to manage oxidative stress, repair damage, and support growth and development. Banaei et al. [74] found that MPs caused physical or oxidative damage to the intestinal epithelium in *Cyprinus carpio*, leading to reduced amino acid absorption and a significant decline in total and globulin plasma protein content. The unchanged TP levels in the exposed group over 45 days may be attributed to the dynamic equilibrium between protein synthesis and degradation rates.

Moreover, previous studies had reported developmental heterogeneity across life stages in freshwater mussels, resulting in differential sensitivity to environmental pollutants [75]. For example, Wang et al. observed lower copper sensitivity in older juveniles compared to newly metamorphosed ones under laboratory conditions [76]. Emerging research further revealed dose-dependent physiological effects of PS-NPs in bivalves (e.g., *Ruditapes philippinarum*) [22]. Our findings were consistent with the present results. The three-way ANOVA showed that MDA levels in *C. plicata* were significantly affected by exposure duration, concentration, and the age × duration interaction, with juveniles exhibiting earlier and more pronounced changes than adults. SOD activity was modulated by age, duration, concentration, and their pairwise or three-way interactions. Notably, juveniles responded significantly at lower concentrations and shorter exposure periods. This divergence was likely due to juveniles’ underdeveloped antioxidant systems during critical gonadal development. TP content was significantly influenced by age and the age × concentration interaction. These results confirm that juvenile mussels exhibit higher sensitivity in MDA and SOD levels to 50 nm PS-NP exposure than adults.

It was demonstrated that PS-NPs elevated MDA levels, induced oxidative damage, and activated antioxidant defenses in *C. plicata* by producing protective proteins (upregulation of SOD but not limited to SOD). However, when concentration and duration exceeded tolerable thresholds, impairment of the antioxidant defense system was observed. Compared to adults, juveniles exhibited higher sensitivity of oxidative stress biomarkers to PS-NP exposure, along with significantly reduced intestinal protein content. Thus, high concentrations (200 μg·L^−1^) of PS-NPs disrupt redox balance and antioxidant defenses in freshwater mussels, potentially compromising their normal growth and development, with a critical risk window identified during their early developmental stages.

### 4.3. Exposure to PS-NPs Affected C. plicata’s Physiological Metabolism

Antioxidant and stress responses to pollutant exposure require substantial energy, necessitating increased physiological metabolism [77]. Respiratory rate (RR) and ammonia excretion rate (AER) are critical indicators of stress adaptation and metabolic health under environmental stress. Elevated RR indicates an increased metabolic rate, while higher AER suggests enhanced protein degradation [78,79]. After 45 days of exposure, the RR and AER of juvenile mussels in the high-concentration group significantly increased compared to J-CK, with no effect in the low- and medium-concentration groups, supporting that RR and AER of *C. plicata* respond in a dose-dependent manner to 50 nm PS-NP exposure. The rise in RR shows *C. plicata* increased its metabolic rate to meet higher ATP demands for antioxidant and detoxification processes. The concurrent AER increase suggested metabolic reprogramming—accelerating damaged protein breakdown and boosting amino acid catabolism to sustain energy balance under oxidative stress. Similarly, Wang et al. [71] reported increased RR and ammonia excretion in *Mytilus coruscus* following 14 days of exposure to 70 nm PS-NPs [76]. Chackal et al. [80] observed that early developmental stages of zebrafish increased oxygen demand to counteract oxidative stress damage. In this study, a three-way ANOVA revealed that mussel RR and AER were significantly affected by age and the age × duration interaction, with significant differences observed between adult and juvenile mussels on days 30 and 45. These findings were attributed to the greater energy reserves in adults, which mitigated PS-NP toxicity. These results further demonstrate that the physiological responses of *C. plicata* to 50 nm PS-NP exposure are developmental stage-dependent.

This study demonstrates that adults maintain metabolic balance via increased RR against NPs, while juveniles enhance RR and AER to alleviate oxidative stress. These adaptive mechanisms may impose additional physiological stress on juvenile mussels, potentially impacting their growth and development.

### 4.4. Exposure to PS-NPs Disturbed Gut Microbiota

In environmental toxicology, the gut microbiota is being increasingly recognized for enhancing the host’s adaptive capacity by regulating physiological processes, including nutrient absorption and energy metabolism [81]. In this study, adult bivalves exhibited the lowest shared OTUs between the control and A-L but significantly higher overlap between the control and A-M/A-H. Low-concentration PS-NPs did not significantly alter adults’ core microbial OTUs, whereas high doses likely suppressed host immunity or promoted the overgrowth of dominant taxa (e.g., Proteobacteria, Firmicutes). Specifically, Bacteroidetes increased markedly in A-M, while Proteobacteria and Firmicutes rose markedly in A-H (Figure 8). These shared OTUs remained high as these taxa pre-existed in controls. Juveniles under medium/high PS-NPs showed similar shared OTUs with J-CK, contrasting adults. Their immature gut microbiota—with lower stability, higher plasticity, and undeveloped immune regulation—may promote uniform response to exposure. Moreover, in adult mussels, α-diversity significantly increased in low and high-concentration exposure groups, whereas it was significantly decreased in the high-concentration exposure group of juvenile mussels. These results indicate that PS-NP exposure notably affects the intestinal microbiota diversity in *C. plicata*. Consistent with our findings, Li et al. [82] reported that *Ctenopharyngodon idella* exposed to environmentally and ecologically relevant concentrations of PS-NPs for 5 days experienced significant gut microbiota changes at the phylum level, disrupting microbial community composition and homeostasis. β-diversity analysis also highlighted significant structural differences in gut microbiota exposed to various PS-NP concentrations. Such microbial diversity and composition changes may impair immune function [41].

Santos et al. [34] highlighted the significance of Firmicutes, Bacteroidetes, Actinobacteria, and Proteobacteria in gut microbiota health, corroborated by the structural changes observed in this study (Figure 8). Our microbial community analyses showed that Proteobacteria abundance significantly decreased in the A-M adult mussel group and all exposure juvenile groups, contrasting with the A-H group. These trends align with previous studies on *Mytilus galloprovincialis*, *Corbicula fluminea*, and zebrafish with NP exposure [21,64,83], where increased Proteobacteria abundance is associated with dysbiosis [84] and decreased abundance can impact immune defense [85]. In this study, the relative abundance of Firmicutes increased significantly in the A-H group and J-M/J-H group, and the Firmicutes/Bacteroidetes ratios also increased. Increased Firmicutes abundance is linked to energy imbalance and obesity risk [86], while changes in Firmicutes/Bacteroidetes ratios signal gut health risks [87,88]. In this study, Fusobacterium abundance was significantly decreased in adult mussels of the A-L and A-H groups. Previous studies suggested that these host-beneficial bacteria help maintain gut microbiota stability by producing butyrate to enhance intestinal metabolism [89]. These results suggest that NPs may cause dysbiosis by reducing beneficial bacteria and increasing harmful ones. A previous study has shown that 0.1 mg/L and 1 mg/L NPs significantly affected the microbial community structure of key taxa in *C. fluminea* [64]. Moreover, 80 nm PS-NPs at environmentally relevant concentrations (0.3, 3 μg/L) strongly disrupted the intestinal microbial community of grass carp, including Proteobacteria, Bacteroidetes, Firmicutes, and Fusobacteria [82].

Furthermore, this study found more significant changes in the microbial community diversity and composition of adult mussels than in juveniles, indicating greater damage in adults. This contradicts hypothesis two. This discrepancy can be influenced by multiple factors, such as the lack of mucosal protection, damage to cilia, and increased intestinal permeability. In contrast, the less developed juvenile mussels, which do not produce mucus, exhibited smaller structural differences in their microbial communities. This study also found that the number of significantly changed bacterial phyla in the exposure groups of adult mussels increases with concentration, whereas the opposite occurred for juvenile mussels. These results demonstrate that 50 nm PS-NP exposure induced dose-dependent alterations in both the diversity and composition of intestinal microbiota in adult and juvenile mussels.

The study demonstrated that PS-NPs significantly impacted the gut microbiota diversity and structure in *C. plicata*. Exposure to PS-NPs triggered major shifts in phyla abundance in both adult and juvenile mussels, depleted beneficial microbiota essential for digestion and immunity, and disrupted the gut microbiota balance. This imbalance elevates the risk of pathogen and toxin cell entry, ultimately affecting the health status of *C. plicata*. The study explored the impact of PS-NPs on freshwater mussels’ intestinal integrity and health across growth stages. While these findings shed light on PS-NPs’ toxicity, further research is needed to fully understand these effects. The 45-day exposure period may not capture all changes in actual environments. Future studies should consider longer exposure times or multigenerational experiments to assess cumulative impacts and potential intergenerational effects. Additionally, in actual environments, NPs are irregularly shaped fibers or particles, which differ from the plastic microbeads used in ecosystem cultivation experiments. These insights will enhance our understanding of the long-term and multigenerational impacts of PS-NPs, as well as their toxic effects in actual environments, supporting better protection and restoration of freshwater ecosystems.

## 5. Conclusions

This study demonstrates that 45-day exposure to environmentally relevant PS-NP concentrations adversely affected the physiological and biochemical activity and intestinal ecosystem balance of *C. plicata*, indicating both dose-dependent and age-related toxicity. Primary responses included oxidative damage to intestinal tissue, characterized by disarray and damage to epithelial cilia, and reduced antioxidant enzyme activity. Juveniles experienced greater physiological stress from PS-NPs than adults. As a compensatory response, *C. plicata* increased respiratory rate, ammonia excretion, and protein turnover. Furthermore, PS-NP toxicity also disrupted the intestinal microbiota. Overall, the study advanced the understanding of NP ecological toxicity in freshwater mussel ecosystems, where significant damage was evident following short-term exposure.

## Figures and Tables

**Table 1:** The outcomes of the three-way analysis of variance (ANOVA) to assess the ages, PS-NP exposure concentrations, and durations of malondialdehyde (MDA) and superoxide dismutase (SOD) activities, as well as total protein (TP) content in *C. plicata*.

Item	MDA		SOD		TP	
	F	*p*	F	*p*	F	*p*
age	2.666	0.109	31.000	<0.001	26.719	<0.001
concentration	11.877	<0.001	5.051	0.004	0.905	0.446
duration	27.086	<0.001	37.708	<0.001	1.877	0.164
age × concentration	1.026	0.389	8.252	<0.001	4.541	0.012
age × duration	8.123	0.001	61.866	<0.001	1.689	0.196
concentration × duration	3.914	0.003	21.267	<0.001	4.418	0.007
age × duration × concentration	2.016	0.082	13.754	<0.001	4.773	0.007

**Table 2:** The outcomes of the three-factor three-way analysis of variance (ANOVA) to assess the age, PS-NP exposure concentrations, and durations of the respiration rate (RR) and ammonia excretion rate (AER) in *C. plicata*.

Item	RR		AER	
	F	*p*	F	*p*
age	592.723	<0.001	132.785	<0.001
concentration	5.371	0.006	1.17	0.331
duration	27.696	<0.001	1.249	0.296
age × concentration	1.977	0.144	1.782	0.163
age × duration	21.076	<0.001	5.148	0.009
concentration × duration	0.982	0.459	0.323	0.922
age × duration × concentration	2.045	0.099	0.898	0.504

## Data Availability

All sequencing data have been deposited to the National Center for Biotechnology Information (NCBI) under the BioProject ID PRJNA1223668 (http://www.ncbi.nlm.nih.gov/bioproject/1223668) (accessed on 14 February 2025). For further assistance or inquiries regarding data access, please contact the corresponding author at 120280164@qq.com.

## Supplementary Materials

The following supporting information can be downloaded at: https://www.mdpi.com/article/10.3390/toxics13050374/s1, Figure S1: Transmission electron microscopy (TEM) image of PS-NPs (scale bar: 100 nm); Figure S2: Fourier transform infrared (FTIR) spectrum of PS-NPs.
