# Supplementary material for: Nanoplastics Elicit Stage-Specific Physiological, Biochemical, and Gut Microbiome Responses in a Freshwater Mussel
Source: Toxics. 2025 May 5;13(5):374. doi: 10.3390/toxics13050374 (PMC12115734; doi:10.3390/toxics13050374)

Fig. S1: Transmission electron microscopy (TEM) image of PS-NPs (scale bar: 100 nm)

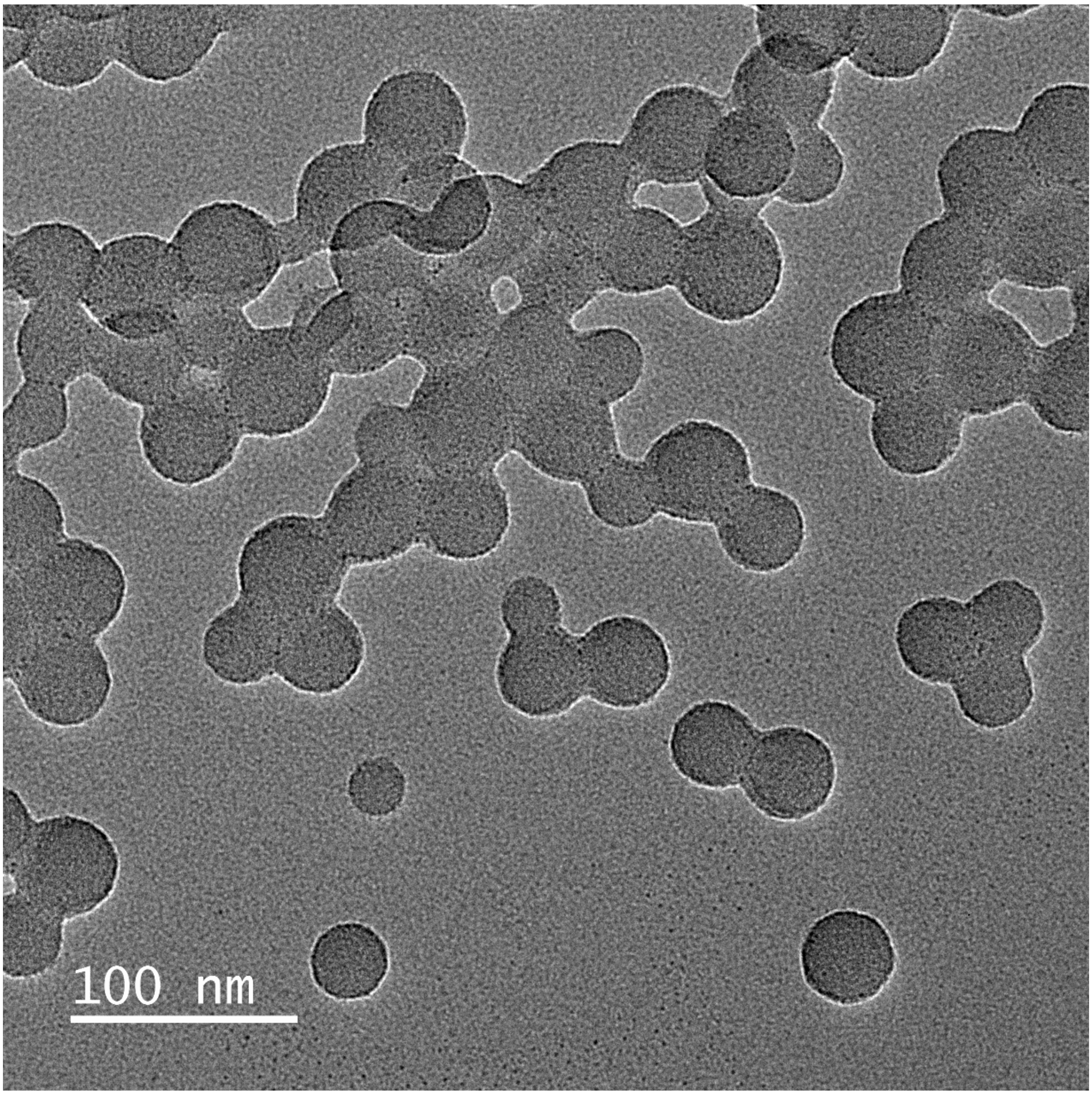

Fig. S2: Fourier transform infrared (FTIR) spectrum of PS-NPs

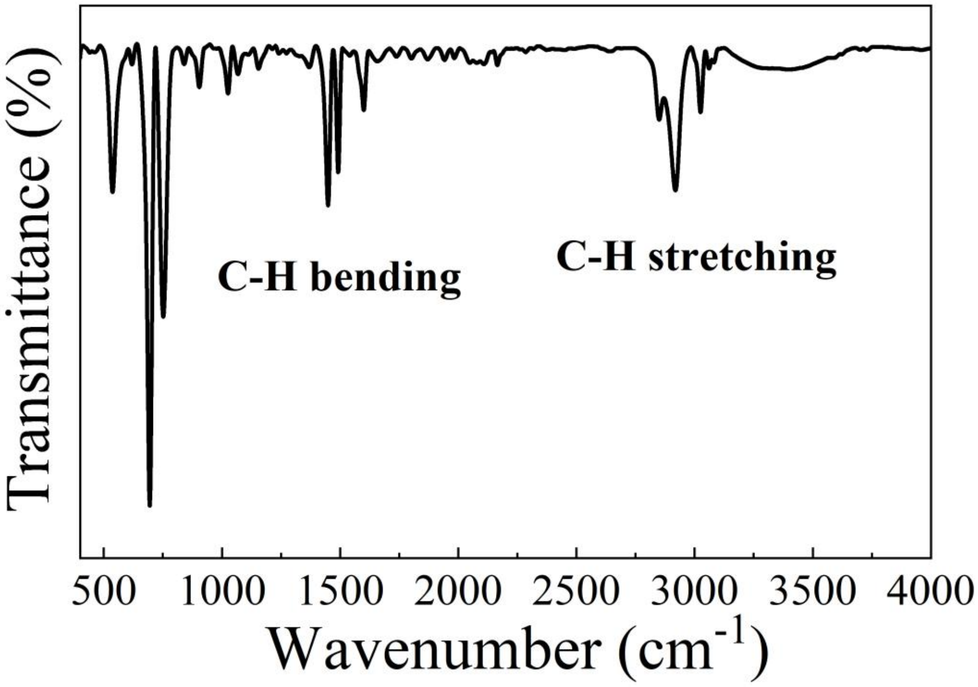

Supplement: Supplementary file 1 [file toxics-13-00374-s001.zip › toxics-3529729-Supplementary material.pdf]
